# Leucine Aminopeptidase, β-Glucosidase and Alkaline Phosphatase Activity Rates and Their Significance in Nutrient Cycles in Some Coastal Mediterranean Sites

**DOI:** 10.3390/md8040916

**Published:** 2010-03-29

**Authors:** Gabriella Caruso

**Affiliations:** Institute for Coastal Marine Environment, Italian National Research Council, CNR, Messina, Italy; E-Mail: gabriella.caruso@iamc.cnr.it; Tel.: +39-090-669-003; Fax: +39-090-669-007

**Keywords:** organic matter decomposition, microbial enzymes, coastal Mediterranean sites, biogeochemical cycles, microbial ecology

## Abstract

In aquatic microbial ecology, knowledge of the processes involved in the turnover of organic matter is of utmost importance to understand ecosystem functioning. Microorganisms are major players in the cycling of nutrients (nitrogen, phosphorus) and carbon, thanks to their enzymatic activities (leucine aminopeptidase, LAP, alkaline phosphatase, AP, and β-glucosidase, β-GLU) on organic polymers (proteins, organic phosphates and polysaccharides, respectively). Estimates of the decomposition rates of organic polymers are performed using fluorogenic compounds, whose hydrolysis rate allow us to obtain information on the “potential” metabolic activity of the prokaryotic community. This paper refers the enzyme patterns measured during recent oceanographic cruises performed in some coastal Mediterranean sites, not yet fully investigated in terms of microbial biogeochemical processes. Mean enzyme activity rates ranged from 5.24 to 5558.1 nM/h, from 12.68 to 244.73 nM/h and from 0.006 to 9.51 nM/h for LAP, AP and β-GLU, respectively. The highest LAP and AP activity rates were measured in the Gulf of Milazzo (Tyrrhenian Sea) and in the Straits of Messina, in association with the lowest bacterioplankton abundance; in contrast, the lowest ones were found in the northern Adriatic Sea. β-GLU was more active in the Straits of Messina. Activity rates were analysed in relation to the main environmental variables. Along the northern Adriatic coastal side affected by the Po river, significant inverse relationships linked LAP and AP with salinity, pointing out that fluvial inputs provided organic substrates for microbial metabolism. Both in the Gulf of Manfredonia and in the Straits of Messina, LAP and AP levels were inversely related with the concentration of nitrate and inorganic phosphorus, respectively. In the Gulf of Milazzo, high cell-specific AP measured in spite of phosphorus availability suggested the role of this enzyme not only in phosphorus, but also in carbon release.

## 1. Introduction

In aquatic environments the bulk of organic matter is a highly heterogenous matrix, composed of labile substrates such as proteins or peptides, simple sugars and fatty acids and more refractory substrates, such as structural carbohydrates or colloids [1]. Organic matter decomposition plays a key role in the functioning of aquatic ecosystems; within this process prokaryotes, including heterotrophic bacteria, are recognised to be major players, thanks to their small size, high biomass and biochemical diversity [2–7]. Particularly, the prokaryotic-mediated enzymatic hydrolysis of organic polymers is generally considered as the first step limiting the transformation of both dissolved and particulate organic matter [1,6,7]. Through the decomposition process, microorganisms regulate nutrient (nitrogen and phosphorus) regeneration and carbon cycling that take place along the microbial loop [2,10]. The monomers released by the hydrolytic process are further utilised by microorganisms themselves to sustain their productive processes; therefore they act both as decomposizers and producers [8,9].

Coastal marine environments receive both autochthonous (phytoplankton excretion, cell lysis, sloppy feeding) and allochthonous (terrestrial) inputs; here bacterial utilization of allochthonous carbon was found to occur independently of primary production, due to the loose coupling between phytoplankton producers and bacterial consumers [11]. Enhanced rates of microbial activities have been measured in these areas, in comparison with pelagic, oligotrophic, ones; in fact, heterotrophic bacteria are able to adapt their metabolic profiles, to better utilize the substrates that are available to themselves [12]. Consequently, a higher variability in the patterns of microbial metabolism than in total bacterial abundances has been found in recent studies [13–17], in response to the supply of both allochtonous and autochtonous substrates.

Despite the importance of coastal environments as sites of particular biogeochemical significance, in the Mediterranean Sea the microbial decomposition played by enzyme activities has been the subject of a few studies, often spatially limited [11,13–25]. Starting from 1995, most researches performed at the IAMC-Messina in the Mediterranean waters have focused on the determination of the activity rates of three enzymes among the most widespread in marine environments, namely leucine aminopeptidase (LAP), β-glucosidase (β-GLU) and alkaline phosphatase (AP). In particular, both LAP and β-GLU are involved in the decomposition of proteins and polysaccharides and therefore in carbon cycling, whereas AP regulates phosphorus regeneration from organic phosphoric esters [7]. LAP is a very widespread enzyme in marine waters, synthesized by bacteria and cyanobacteria, phytoplankton and zooplankton; it is involved in the decay of particulate matter composed of living organisms or nonliving materials, such as faecal pellets *etc.* [17,26]. High levels of aminopeptidase activity are generally recorded in the surface layers, mainly due to the supply of labile, freshly produced, organic substrates [16]. Protein-containing particles are abundant in the sea and in particular, in the coastal environment since proteins are a major component of biogenic organic matter [27]. β-GLU is an enzyme specific for the hydrolysis of cellobiose contained in polymers such as cellulose and mucopolysaccharides; β-GLU values are often related to Chl-*a* and POC, which are substrates rich in these organic compounds [18,19]. This enzyme is mostly associated with heterotrophic bacteria, although also zooplanktonic species are reported to possess this activity [6]. AP represents a key enzyme in natural environments, since it allows P regeneration as dissolved inorganic orthophosphate from phosphoesters [28]. In addition to bacteria, phytoplankton, phototrophic prokaryotes and protozoa may contribute to the pool of freely dissolved AP in the sea [29]. Usually AP is an inducible enzyme, being synthesized at low levels of PO_4_ and repressed when P becomes available; therefore it has been suggested as a P-deficiency index. However, bacteria can also produce AP to supply their metabolism with organic carbon substrates which are released as final products of phosphoester hydrolysis. Therefore AP could be a constitutive enzyme or produced by C-limited bacteria in response to insufficiently available organic carbon concentration and it may contribute to organic P and C cycling simultaneously [29,30].

The aim of this paper is to contribute to current knowledge of the functioning of some coastal Mediterranean ecosystems, in terms of microbial biogeochemical processes; the data until now available in these sites on the microbial decomposition played by enzyme activities have been summarized here, in order to assess their ecological role within the carbon and phosphorus cycles.

## 2. Results and Discussion

### 2.1. Northern Adriatic Basin

During February 2008, the Adriatic waters displayed temperatures ranging from 7.76 to 11.68 °C and salinity values varied between 34.32 and 38.55. Table 1 shows the mean value and ranges of variation of enzyme activity rates. Enzyme patterns showed high AP activity rates; discrete values of LAP and β-GLU were also found.

**Figure 1:**
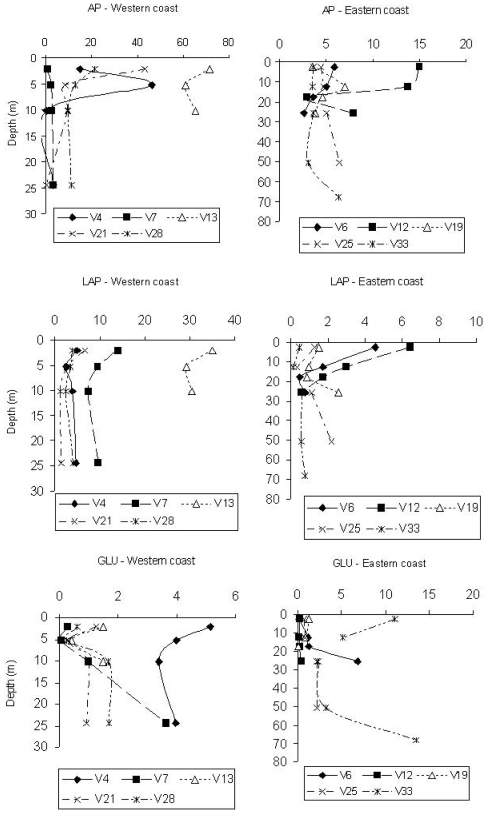
Northern Adriatic Sea. Vertical profiles of enzyme activity (in nM/h).

Spatially, maximum AP values were found at stations V13 and V4, belonging to transects C and A, respectively. In contrast, minimum values were detected at station V7, belonging to transect B. The Eastern side was characterised by AP activity rates on average ¼ lower than those measured along the Italian coast (Figure 1). AP activity levels decreased south of the Po river, suggesting a lower amount of organic phosphates or the availability of P.

Vertical AP profiles (Figure 1) showed higher enzyme values at surface or sub-surface depths, such as at 5 m at station V4; some activity peaks, however, were found at the bottom of the stations V25 and V33, along the Eastern coast.

LAP rates were higher on the Western Adriatic side, especially at stations V13 and V7, and lower at stations V21 and V28 (transects D and E). The Eastern stations displayed enzyme levels about 1/3 lower than those measured at the Western ones; minimum values were found at station V33 (Figure 1). LAP levels were related to organic matter inputs coming from the Po river; in fact, station 13 was characterised by the highest mean POC and Chl-*a* values (41.54 and 1.45 μg/L, respectively), while the lowest ones were found at station 33 (3.48 and 0.152 μg/L, respectively).

Integrated LAP values obtained for each station (not reported in figure) showed that enzyme levels followed an increasing pattern from station V33 northwards to station V12 (0.507 to 2.943 μmol/m^3^/h) along the Eastern side, and from station V4 southwards to station V13 (4.01 to 31.50 μmol/m^3^/h) along the Western side, in agreement with the circulation pattern of the water masses within the Northern Adriatic basin.

Vertical LAP profiles (Figure 1) showed the decrease of enzyme activity levels from surface to bottom along the coastal side affected by the Po river inputs, while at stations V19, V25 and V33, located along the Eastern coast, peaks at greater depths were observed. Here, at the bottom, POC values were slightly higher than those found in the nearest upper layers (3.82, 3.63, 3.12 μg/L *vs.* 3.32, 3.08 and 1.76 μg/L, respectively), suggesting the sinking of labile matter.

The highest β-GLU activity rates were measured at stations V33 (transect E), V4 and V6 (transect A); in contrast, minimum values were detected at station V12 (Eastern side) (Figure 1). The highest activity rates were found along the Western side; particularly, at station V4 the lowest amounts of Chl-*a* and POC were measured (0.090 and 3.14 μg/L, respectively). From the integrated β-GLU values obtained for each station (not shown), a decreasing gradient was observed from station V4 southwards to station V13 (4.117 to 1.130 μmol/m^3^/h) on the Western coast, and from station V33 northwards to station V12 (7.055 to 0.271 μmol/m^3^/h) on the Eastern one. This latter side was characterised by enzyme values about 1.3 times higher than those measured on the Western one.

Vertical β-GLU profiles (Figure 1) mostly followed an increasing trend with depth; generally, at the Eastern side stations a higher activity was found at lower depths (stations V6 and V33), suggesting the presence of more refractory substrates, probably of polysaccharidic origin. This was also in agreement with the detection at station 33 of the lowest concentrations of Chl-*a*, as an indicator of fresh organic matter.

Pooling all the data collected from the Western (n = 20) and the Eastern stations (n = 22) separately, AP rates were negatively affected by salinity (r = −0.811, P < 0.01) along the Western side; by temperature (r = −0.684, P < 0.01) along the Eastern one. LAP activity rates showed negative correlations with salinity (r = −0.771, P < 0.01) along the Po coast as well as along the Eastern side (r = −0.833, P < 0.01). LAP correlated inversely with temperature (r = −0.799, P < 0.01) along the Eastern side. No significant correlations were calculated between β-GLU and temperature or salinity. On the Western side, the statistical relationships linking enzyme activity rates with POC and Chl-*a* concentrations were generally more significant (for LAP: r = 0.901 and 0.972; for AP: r = 0.921 and 0.952, respectively) than those found on the Eastern one (for LAP: r = 0.904 and 0.832; for AP: r = 0.784 and 0.729, respectively).

### 2.2. Gulf of Manfredonia

During May 2003, temperature values ranged from 12.94 to 19.98 °C, salinity ranged from 37.29 to 38.77. Table 2 shows the mean value and ranges of variation of enzyme activity rates.

In this ecosystem, again AP was measured at high levels. LAP values were higher than those recorded in the Northern Adriatic basin, while lower β-GLU values were found.

**Figure 2:**
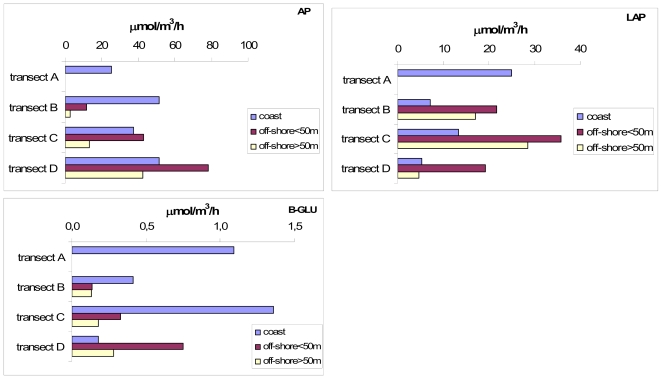
Gulf of Manfredonia. Integrated and depth-normalised values of AP, LAP, β-GLU obtained for each examined transect, showed for coastal stations (<50 metres) and the most off-shore ones, further separated into surface (<50 metres) and deeper (>50 metres) layers.

The particular haline structure observed during middle spring in the Gulf of Manfredonia revealed the presence of a frontal system, located around the 50 m depth bathymetric line, which separated coastal from open sea waters. The distribution of enzyme activity values within this basin reflected this separation between the most coastal stations and the most off-shore ones; at these latter stations, two different layers, the surface (<50 m) and the deep (>50 m) ones, were distinguished (Figure 2).

AP showed increasing activity levels from transect B to D; the activity of this enzyme increased moving southward, especially at the most off-shore stations, both at surface and deep layers, and reached a peak in transect D. LAP was most active at both transects C and B, particularly at the offshore sites. High proteolytic activity was also found at the northern transect (A). B-GLU showed the highest activity levels at transects C and A. Similarly to AP, this enzyme activity increased at the offshore sites moving southward.

Pearson correlation coefficients calculated on all the data (n = 46) showed the occurrence of significant relationships between the dissolved oxygen content and AP rates (r = 0.279, P < 0.05, respectively). Both AP and β-GLU were positively related to total bacterioplankton abundance (r = 0.39, 0.326, P < 0.01). β-GLU correlated inversely to salinity (r = 0.563, P < 0.01), and directly to both temperature (r = 0.516, P < 0.01) and Chl-*a* content (r = 0.431, P < 0.01); this latter relationship was consistent with the release of organic substrates rich in polysaccharides during the productive process.

### 2.3. Gulf of Milazzo

In this area, during December 2002, temperature ranged from 14.21 to 17.56 °C and salinity from 37.82 to 38.59. In February 2003, temperature ranged from 13.79 to 14.42 °C and salinity from 37.61 to 38.62. The mean values and the ranges of variation of enzyme activity rates are reported in Table 3.

**Figure 3:**
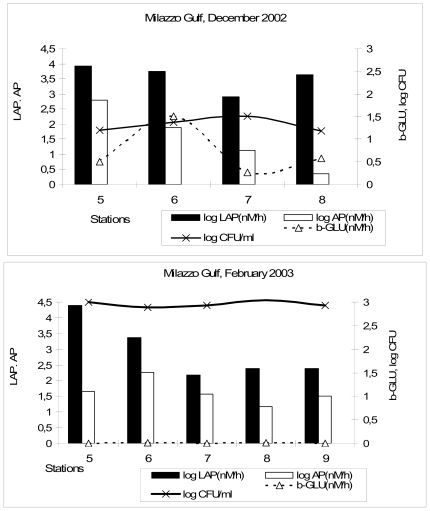
Gulf of Milazzo. Enzyme activity rates measured along the examined coastal (station 5) to offshore (station 9) transect.

β-GLU and AP were significantly higher (P < 0.01) in December compared to February. Enzyme patterns displayed a general decreasing trend with increasing distance from the coast in both the examined periods (Figure 3). Due to water column stratification, the highest LAP and AP levels, as well as the highest autotrophic (Chl-*a*) and culturable heterotrophic (CFU) biomasses, were confined in this layer, where the metabolic activities correlated positively with temperature (r = 0.997, P < 0.01; 0.954, P < 0.05; 0.993, P < 0.01, n = 10, for LAP, β-GLU and AP respectively). The stratified water structure was also reflected in the highest coefficient of variations [calculated as standard deviation/mean*100] found in this area for heterotrophic (94.96) and autotrophic (50.53) biomasses. Chl-*a* concentration and heterotrophic bacteria were significantly correlated to each other (r = 0.758, P < 0.01) and positively to temperature (r = 0.734 and 0.941, P < 0.01, respectively). Within the Deep Chlorophyll maximum (DCM), the contribution of phytoplankton to the release of AP was suggested by the close relationship (r = 0.938, n = 5, P < 0.05) detected between AP and Chl-*a*.

In February 2003, when water column remixing started, values of about two orders of magnitude higher than those previously found were noticed for LAP and bacterial heterotrophic density. Chl-*a* content reflected a similar increasing trend, with higher values measured in February than in December (Table 3). The phytoplankton growth was supported by high ammonia (0.12–6.05 μM) and inorganic phosphate (0.1–0.92 μM) concentrations available in this period. In contrast, AP and β-GLU activity values were lower than in the first sampling, ranging from 0.256 to 463.96 nM/h and from 0.0011 to 0.015 nM/h respectively. Peaks of AP and Chl-*a* shifted from surface towards intermediate depths, following the increasing trend of temperature towards the bottom, while LAP activity and heterotrophic biomass distribution were not affected by the change in hydrological condition, being always concentrated in surface layers. Within the DCM, all the bacterial enzyme activities were significantly correlated (r = 0.989, P < 0.01; 0.952 and 0.909, n = 5, P < 0.05, for LAP-AP, LAP-βGLU and β-GLU-AP, respectively).

### 2.4. Straits of Messina

During the study period (June 2003–March 2004), temperature varied between 14.01 and 27.22 °C, whereas salinity ranged from 37.52 to 38.94. Table 4 shows the mean value and ranges of variation of enzyme activity rates.

On a spatial scale, different patterns of enzyme activities were detected: in fact, at station Scaletta, the mean LAP, β-GLU and AP activity rates were higher than those measured in the other sites, providing a significant N, C and P source, respectively. Minimum enzyme activity levels were generally measured at station Pellaro (Table 4).

**Figure 4:**
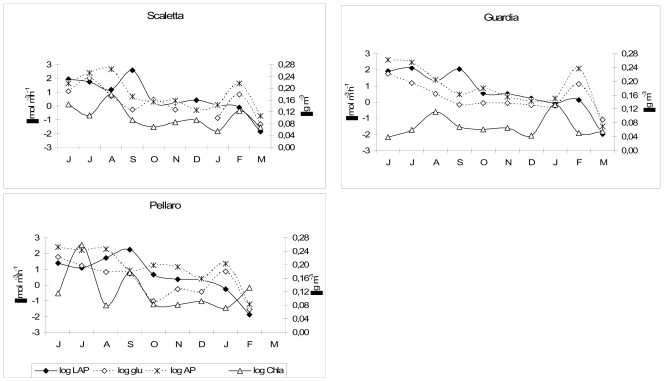
Straits of Messina. Integrated and depth-normalised values of LAP, β-GLU, AP and Chl-*a* obtained at stations Scaletta, Guardia and Pellaro.

Enzyme patterns underwent temporal variations (Figure 4). The highest LAP values were detected in summer, with a peak in September, while lower values, never exceeding 10 nM/h were measured in the remaining months. High β-GLU values were measured in June–July at station Scaletta; this enzyme prevailed in correspondence to the DCM layer (not shown in Figure), suggesting a preferential microbial attack on polysaccharides. AP was particularly active in autumn at station Pellaro.

Culturable heterotrophic bacteria were mostly lower than 100 CFU mL^−1^, increasing towards the bottom in July-August, because of the enrichment of intermediate waters with monomers or dissolved compounds produced by hydrolysis.

As shown in Figure 4, in summer (June to September) high enzyme activity rates were measured, suggesting the occurrence of degradative processes driven by heterotrophs; Pellaro was also characterised by high Chl-*a* concentrations, indicating high productive processes. From October to December (autumn period) low values of autotrophic biomass and microbial enzyme activities were measured everywhere. In winter (January to March), both autotrophic biomass and heterotrophic activities were enhanced in Guardia; among the examined enzymes, AP and β-GLU displayed high activity rates, resulting in active P regeneration and polysaccharide decomposition, respectively. The nutrient consumption operated by autotrophs during autumn, due to phytoplankton growth, justified the stimulated production of enzymes involved in nutrient cycling.

### 2.5. Main Considerations on Enzyme Patterns and Their Biogeochemical Significance

Microbial assemblages play a dominant role in the transformation and mineralization of organic matter in the marine environment [1,6,9]. Dissolved organic polymers are the major reservoirs of Carbon and nutrients in the waters; while they are produced by different planktonic sources and biological processes, as well as introduced from land and fluvial inputs, they are consumed and recycled mainly through bacterioplankton. Therefore, bacterial metabolism regulates the cycling of biogenic elements [31]. Before the present study, the patterns of microbial activities involved in biogeochemical cycles were fully unknown in the waters of the Gulfs of Manfredonia and Milazzo, as well as in the Straits of Messina. The enzyme measurements reported here are the first available for these coastal areas, in contrast to some other Mediterranean sites (Ligurian Sea [23]; northwestern Mediterranean, DYFAMED station located at the western part of the Ligurian Sea [21,22,24,25]), where extensive studies were previously performed. Even in the Northern Adriatic basin, where most of the studies dealt with the appearance of mucilages [17,20,32,33], only a few investigations focused on the dynamics of microbial assemblage (abundance and activity) on a wide spatial (*i.e.*, basin) scale similar to that considered in this study.

The use of fluorogenic substrates to estimate enzyme activity rates provide a “potential” estimate of activity only; nevertheless, this potential information is of great ecological significance, as it contributes to knowledge of the microbial potentialities on the organic matter pool and therefore to better define the role played by microorganisms in the functioning of aquatic ecosystems.

The measured enzyme activity rates, converted into nanograms of Carbon and Phosphorus potentially released from the substrates, are summarised as the mean values obtained for each area and reported in Table 5. LAP and AP values increased from the Northern Adriatic Sea to the Straits of Messina, through the Gulf of Manfredonia; β-GLU was at the lowest levels in the Gulf of Milazzo (February 2003). Enzyme values detected in the study areas fall within a range similar to that detected in other Mediterranean coastal environments [1,15,16,19,21,32,33].

This comparative investigation pointed out the widespread occurrence of LAP and AP activities in the studied coastal areas, confirming the high potential of the microbial community to degrade preferentially proteins and organic phosphoric esters, respectively. In the waters of the North Adriatic Sea, this result agreed with the *in vitro* determination of enzyme profiles, which showed the expression of peptidase and phosphatase by a large (60%) percentage of bacterial isolates in all the seasons [17].

The microbial enzyme activity patterns found in this study were characterised by high variability both in space and time. Analysis of variance (ANOVA) revealed significant spatial differences among the different ecosystems in terms of their LAP, β-GLU and AP values (F: 29.74, 52.72, 5.11, P < 0.01, respectively).

The values of Michaelis-Menten constant (K_m_), reported in Table 5, suggested a significantly (p < 0.01) lower substrate affinity of LAP in the Gulf of Milazzo (February 2003) compared to the highest affinity measured in the Gulf of Manfredonia. Conversely, the Gulf of Milazzo (February 2003) was characterised by a much higher substrate affinity of β-GLU with respect to that measured in the Straits of Messina. A low K_m_ value found for AP in the Straits of Messina indicated that in this area the microbial community could use a small amount of substrate effectively, while the high K_m_ found in the Gulf of Milazzo (December 2002) suggested the need for high substrate concentration to achieve maximum reaction velocity. High substrate affinity is considered a strategy to support bacterial growth under low nutrient conditions [34].

The enzymatic ability measured in coastal environments allows to estimate the amount of nutrients that can be potentially mobilised and exported to the open sea. The potential remineralisation rate of phosphorus by AP was 9.43 μg P/dm^3^/day in the Northern Adriatic Sea and 26.34 μg P/dm^3^/day in the Gulf of Manfredonia; the same enzyme activity was estimated to contribute to the release of 80.34 μg P/dm^3^/day in the Straits of Messina, taking into account the entire period. In the Gulf of Milazzo, AP activity potentially contributed to the remineralisation of 182.1 and 46.51 μg P/dm^3^/day in December and in February, respectively.

LAP potentially accounted for the release of 1.76 μg of dissolved organic N/dm^3^/day in the Northern Adriatic Sea, while this amount increased in the Gulf of Manfredonia and in the Straits of Messina, where we estimated that the dissolved organic N potentially mobilised per day was 6.55 μg and 17.27 μg N/dm^3^/day, respectively. In the Gulf of Milazzo, LAP activity potentially released 1.80 μg and 1.87 mg of dissolved organic N/dm^3^/day in December and in February, respectively.

Comparing the different ecosystems, the highest potential mineralization rate of P by AP, calculated on a daily scale, was found in the Gulf of Milazzo (December 2002) and in the Straits of Messina; LAP potentially contributed to the release of high amounts of N in the Gulf of Milazzo (February 2003) and in the Straits of Messina. These estimates suggested the highest potentiality of microbial assemblages inhabiting these two environments in decomposing organic polymers. The expression of high decomposition rates in these areas confirmed the importance of heterotrophic metabolism in oligotrophic Mediterranean ecosystems, in agreement with other studies [9,23,35].

Moreover, because of the different substrate analogues used for measuring enzyme activity rates, the reciprocal ratios among the single ectoenzymatic activities (LAP/β-GLU, LAP/AP) were also calculated to better characterise and compare the different ecosystems under study (Table 6).

Variations in the relative activities of different enzymes can also indicate geographical variations in the modalities of bacterial nutrition [36]. High LAP/β-GLU ratios provide information on the flux of organic matter preferentially through proteins, while low ratios indicate a preferential flux of organic matter through polysaccharides. The highest values of this ratio were reached in the Gulf of Milazzo (February 2003), because of the lowest β-GLU levels; high values were also observed in the Gulf of Manfredonia and Milazzo (December 2002), two areas where similar low β-GLU activity rates were measured. High LAP/β-GLU values are generally found in many temperate coastal environments [1,18,21,23,32], suggesting a higher potential degradation of proteins compared to that of polysaccharides. LAP values significantly (from 5 to 300 times) higher than β-GLU were also detected during recent investigations in the Adriatic Sea; on the other hand, the greater amount of LAP compared to β-GLU may be explained by the capability of released amino acids to provide a source of both C and N for bacteria [18].

LAP/AP ratios provide insights on the relative importance of N mobilization compared to P mineralization. Sala *et al.* [37] suggested the use of AP/LAP ratio for evaluating P *versus* N limitation of microbial communities “*in situ*”. In our study, LAP/AP ratios were generally lower than 1, with a minimum value in the Gulf of Milazzo, during December 2002; this was the consequence of AP activity higher than LAP and suggested the greater importance of P mineralization. An exception was the Gulf of Milazzo during February 2003, when LAP prevailed over AP, so resulting in the highest LAP/AP ratio, and enhanced N mobilization.

In addition, in order to assess whether enzyme activities were mostly cell-associated, cell-specific activity rates were calculated through normalisation of the enzyme activities to the bacterioplankton abundance. For AP, which is synthesised by both phytoplankton and bacteria, cell-specific AP rates were also normalised to the concentration of Chl-*a*, as a proxy of phytoplankton biomass. Scaling enzyme activity rates to the cell abundance allows to compare different environments for their degradation capability; nevertheless, this elaboration has some limitations, because a percentage of cells might be dormant or dead, different microbial groups could express different activity levels, and have intra-specific differences [12,17,38]. Therefore, for this approach we assumed that all the microbial cells had similar activity levels. Specific LAP *per cell* increased from the Northern Adriatic basin to the Gulfs of Manfredonia and Milazzo (December 2002); increases of about three times were found in the Straits of Messina, and values reached levels about four orders of magnitude higher in the Gulf of Milazzo (February 2003).

Cell-specific GLU ranged from the lowest activity values measured in the Gulf of Milazzo (February 2003) to the highest ones observed in the same area in December 2002 and particularly in the Straits of Messina; in these oligotrophic sites (*i.e.*, characterised by low Chl-*a*, Table 6), the presence of microorganisms with high potentiality in decomposing refractory material was confirmed. In the Southern Tyrrhenian Sea [35], values of cell-specific LAP activity ranged from 6.5 to 23.1 amol/cell/h, while cell-specific β-GLU values ranged from 0.16 to 0.63 amol/cell/h (*i.e.*, corresponding to 0.47–1.66 fg C/cell/h and 0.011–0.45 fgC/cell/h for LAP and β-GLU, respectively).

Cell-specific AP values normalised to bacterioplankton abundance showed that the Gulf of Milazzo (December 2002) was the site with the most active bacterial community in processing organic phosphates. In addition, the Straits of Messina and the Gulf of Milazzo (February 2003) exhibited also high AP *per cell*, normalised to bacterioplankton as well as to phytoplankton biomass. As the specific AP activity normalised to Chl-*a* has been suggested to be a good indicator of P-limited algal growth [39], this result indicated that phytoplankton reacted to this condition producing AP with higher specific activity. Cell specific AP values above 200 nmol/μg Chl-*a*/h, corresponding to 6.20 μg P/μg Chl-*a*/h, were obtained in areas of the central Atlantic Ocean with P-deficiency [40].

Except for the Northern Adriatic Sea, cell-specific enzyme activities calculated in this study were higher than those reported in other coastal Mediterranean sites, such as in the Ligurian Sea [23], where mean cell specific LAP and β-GLU were, on average, 1.8–3.96 fg C/cell/h and 0.072 fg C/cell/h, respectively, or in the NW Mediterranean (DYFAMED station) [24], where mean cell specific AP was 0.062 fg P/cell/h. Cell-specific LAP and AP activity rates were particularly high in the Straits of Messina and in the Gulf of Milazzo, where the bacterioplankton abundances (mean value: 10^7^ cells/l) were about two orders of magnitude lower than those found in the Northern Adriatic Sea. Therefore, high rates of cell-specific activity suggested the presence of a fraction of highly metabolically active bacteria able to release enzymes, rather than the whole bacterial community [41].

Furthermore, it is important to note that, although only cell-associated enzyme activities have been assumed to be of ecological significance [42–44], the contribution of free, dissolved enzymes, to the total enzyme activity as another important source of enzymes in all the studied ecosystems cannot be excluded, as documented by other research performed in coastal [21] as well as in oceanic [45] areas. This could explain why significant relationships between enzyme activities and biotic components were not always detected.

As enzyme activity can be considered the initial response of the microbial community to environmental changes [1,21], attention was also paid to the environmental abiotic (physico- chemical) variables which were responsible for the observed variability in potential enzymatic activity patterns. In the Northern Adriatic Sea as well as in the Gulf of Manfredonia, salinity was the main environmental factor which affected enzyme patterns. Significant negative correlations between both LAP activity and salinity values, suggested the importance of fluvial input in providing allochthonous sources for microbial metabolism. The stratification of the “plume” outflow over the underlying waters was responsible for the local confinement of the trophic supply in the surface layer; this distribution was reflected by the vertical profiles of LAP and AP, showing a decreasing trend with increasing depth, similarly to what observed during previous studies [14,16]. The significant relationship of LAP and AP activity rates with POC found in the Northern Adriatic basin suggested that microorganisms responded to the available organic substrates stimulating specifically enzyme production.

In the Gulf of Manfredonia and in the Straits of Messina, the hydrological circulation patterns acted as the main factor regulating the enzyme distribution. In the Gulf of Manfredonia, Apulian coastal waters became enriched with organic matter coming from the Northern Adriatic basin, and further transported these organic inputs to the most southern transects; there, high AP and LAP activities were found within the first 50 m depth of the most off-shore stations. The coastal to offshore increasing trend followed by LAP activity rates supported the hypothesis that probably the particular circulation of water masses spread labile matter, not fully degraded in the coastal area, towards the offshore one. In the Straits of Messina, intense hydrodynamism such as that related to upwelling and cyclical changes of both Tyrrhenian and Ionian water masses, was supposed to modulate the nature and composition of organic inputs, determining, in turn, changes in the enzymatic spectra of microorganisms.

Changes in the water column structure (*i.e.*, water stratification in late autumn and mixing in winter) were responsible for the temporal variations in the rates of organic matter processing recorded in the Gulf of Milazzo. When the water column was stratified, enhanced activity rates towards more refractory compounds, as shown by β-GLU values, were found. During water mixing, high LAP values suggested that the decomposition process was more active on newly produced material; this was also confirmed by the high Chl-*a* content.

Enzyme activity patterns reflected the diversity of biogeochemical features among the examined sites. In fact, we found distinct patterns of enzyme activities in each ecosystem and there was no consistent trend in the enzyme expression in relation to nutrient concentrations. In the Straits of Messina, the inverse relationship between LAP and nitrogen nutrients, observed during autumn at all the stations (LAP *vs.* NO_3_, r = −0.679, −0.749, −0.768, n = 12, P < 0.05 at Scaletta, Guardia and Pellaro, respectively), as well as during summer and winter at Scaletta station (LAP *vs.* NO_3_, r = −0.59, −0.627, n = 12, P < 0.05), led us to hypothesise a regulation of microbial attack on organic substrates by N rather than P. Also in the Gulf of Manfredonia, LAP was negatively related to NO_3_ (r = −0.259, n = 46, P < 0.05).

Concerning AP, the patterns observed in the present study indicated that the expression of AP was not always regulated by the concentration of its end-product; in fact, a significant inverse relation between AP and PO_4_ concentration was observed in the Gulf of Manfredonia (r = −0.336, n = 46, P < 0.05) and in the Straits of Messina (r = −0.658, n = 40, P < 0.05, Pellaro station during autumn) only. In the Northern Adriatic basin, the lack of a significant inverse relation between AP and PO_4_ (mean value 0.06 μM) could depend on the internal P stored inside the cells, as shown by Sebastian *et al*. [46]. Conversely, in the Gulf of Milazzo the synthesis of high cell-specific AP activity in spite of P availability (mean value: 2.99μM) could probably be a strategy adopted by the microbial community to get not only a Phosphorus but also a Carbon source, similarly to what reported by other Authors [30,38] at meso- and bathypelagic depths in oceanic and sub-tropical Atlantic waters.

## 3. Experimental Section

### 3.1. Study Areas

#### 3.1.1. Northern Adriatic Basin

The Adriatic Sea is an elongated basin, with its major axis in the Northwest-Southeast direction, located in the Central Mediterranean, between the Italian peninsula and the Balkans. The Northern Adriatic basin is a semi-enclosed basin with particular hydrological conditions which determine the occurrence of periodic distrophic crises and mucilage phenomena. This ecosystem is characterised by its shallow depth and the anticyclonic circulation which affects the distribution of the organic matter inputs mostly coming from the Po river and run-off; frontal systems are frequently found, which separate the most diluted waters of fluvial origin from the offshore, more haline, ones [47].

#### 3.1.2. Gulf of Manfredonia

The Gulf of Manfredonia is a basin located in the western part of the south Adriatic Sea south to the Gargano National Park (Apulian cost). It represents a transition zone between the central and southern Adriatic basins, and its waters show characteristics typical of the Ionian Sea such as oligotrophy [48]. This semi-enclosed basin is a complex coastal area under the potential threats of various anthropic activities, such as those deriving from industrial, agricultural, harbor and urban wastes [49]; organic loading from urban and industrial settlements determine the nutrient enrichment of waters.

#### 3.1.3. Gulf of Milazzo

The Gulf of Milazzo is a coastal ecosystem located along the Tyrrhenian Sea, in the north-eastern part of the Sicily (Italy); it receives in its more western part confined and localised organic matter inputs in correspondence of river outflows. As a consequence of the supply of dissolved and particulate substrates, a tendency of this area towards the eutrophy has been observed during the past [50]. Unlike the hydrological and biological parameters, bacterial dynamics and metabolism in this ecosystem are still unknown.

#### 3.1.4. Straits of Messina

Located at the centre of the Mediterranean Sea, the Straits of Messina connect two adjacent basins, the Ionian and the Tyrrhenian Seas, with different oceanographic properties: Tyrrhenian surface waters, which are warmer and have low salinity, spread into Ionian waters, which are characterised by low temperature and high salinity values [51]. The condition of transitional and mixing area results in a great complexity of this ecosystem in terms of hydrological structures, even on a short spatial scale (3.2 Km). Since ancient times [52], the Straits have been investigated concerning their upwelling phenomena and tidal movements. While a considerable body of literature has focused on the influence of high hydrodynamism over biological components and productive processes [53,54], a few interest [55] has been addressed to the microbial community present in the waters and to its ecological role.

### 3.2. Study Surveys and Research Objectives

The surveys had similar sampling strategies and methodological approaches, although their study objectives were slight different.

#### 3.2.1. Northern Adriatic Sea

**Figure 5:**
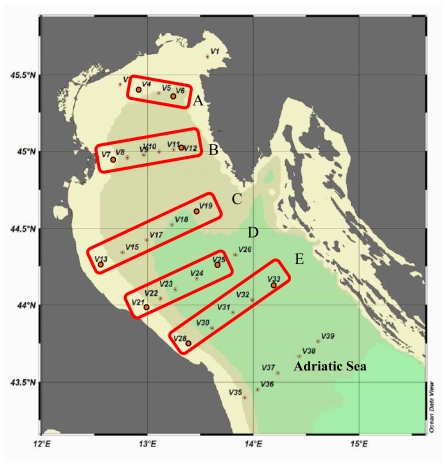
Northern Adriatic Sea. Sampling stations (red spots) located along five transects (A to E).

In the framework of the VECTOR Project, a study was performed in the Northern Adriatic basin, in order to determine the role of the continental shelf in the flux of Carbon across this ecosystem. In February 2008, an oceanographic cruise (“VECSES 1” Cruise) was carried out in a wide area from the Trieste Gulf to Ancona coast on board of the R/V “Urania” of the Italian National Research Council. A total of 15 stations were sampled, located across five coastal to offshore transects (A to E); among these, we selected five stations close to the western, Italian, coast, most affected by Po river inputs and five stations close to the eastern coast (Figure 5).

#### 3.2.2. Gulf of Manfredonia

A sampling cruise was carried out during late spring (May 2003) in the Gulf of Manfredonia by the R/V “Urania”, in the framework of the SAMCA 3 (System Approach to Mediterranean Coastal Areas) research survey. The investigation aimed at assessing the role of microbial mechanisms involved in nutrient cycling in sustaining the productivity of the entire coastal area and adjacent zone.

**Figure 6:**
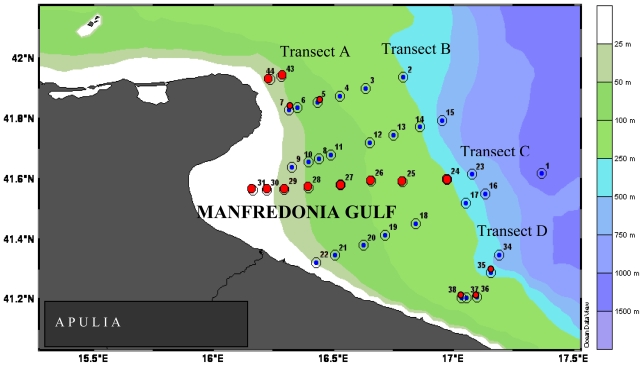
Gulf of Manfredonia. Sampling stations (red spots) located along four transects (A to D).

A total of 129 water samples were taken from 36 hydrological stations at different depths, comprised between 0.5 m and 180 m, located along six transects orthogonal to the shoreline. Among the collected samples, 46 coming from 15 stations located along A, B, D and E transects were selected for the microbiological study (Figure 6).

#### 3.2.3. Gulf of Milazzo

As a part of the SAMCA (System Approach in Marine Coastal Areas) Project Cluster 10–MIUR, devoted at assessing the environmental quality of coastal Sicilian areas, the distribution and variations in extracellular enzymatic activity and bacterial density in the Gulf of Milazzo were investigated, together with the phytoplankton biomass.

**Figure 7:**
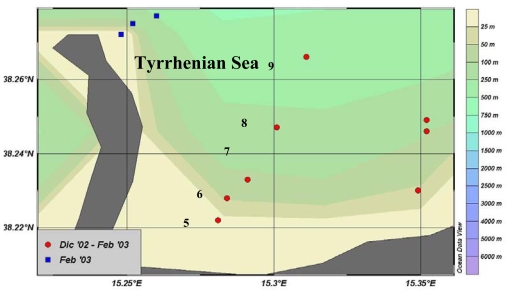
Gulf of Milazzo. Sampling stations (5 to 9, red spots) located along a coastal to offshore transect.

Two oceanographic cruises (December 2002 and February 2003) were performed by the R/V Luigi Sanzo of the IAMC-CNR, during late autumn and winter periods, as representative of different hydrological conditions (water stratification and mixing, respectively). Seawater samples were collected by a rosette sampler at selected depths (surface; above the DCM; at the DCM; below the DCM) across a coastal-offshore transect located in the central-western section of the Gulf (Figure 7).

#### 3.2.4. Straits of Messina

**Figure 8:**
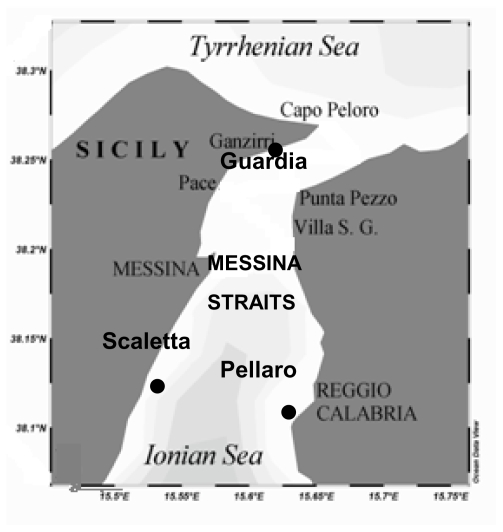
Straits of Messina. Sampling stations: Scaletta and Guardia (on the Sicilian side) and Pellaro (on the Calabrian side).

From June 2003 to March 2004, three coastal sites of this ecosystem were repeatedly sampled, two of which (Scaletta and Guardia) located along the Sicilian side and the other one (Pellaro) on the Calabrian side (Figure 8). Samplings were performed by the R/V Luigi Sanzo of the IAMC-CNR, at four different depths: surface layer, and above, within and below the DCM layer.

The temporal evolution of some microbial activities involved in C and P cycles was investigated in order to obtain a picture of biological processes occurring in the area; particularly, temporal changes occurring in the microbial decomposition of proteins, polysaccharides and organic phosphates were related to the main environmental variables (temperature, salinity, concentrations of nutrients and Chl-*a*) and to the hydrodynamic regimen.

### 3.3. Water Sampling and Treatment

For enzyme measurements, water samples were drawn from different depths of the water column using 10-L acid rinsed Niskin bottles; volumes of 200 mL were kept into sterile containers and either immediately processed on ships or stored at +4 °C for subsequent analysis in laboratory, performed within 2 hours of samplings.

Microbial extracellular enzyme activities were determined according to the Hoppe’s multi-concentration method [56], by adding to 10 mL water subsamples increasing amounts of the specific fluorogenic substrates L-leucine-7-amido-4-methylcoumarin hydrochloride (Leu-MCA), 4-methyl-umbelliferyl (MUF)-β-glucoside and MUF-phosphate for LAP, β-GLU and AP, respectively. Concentrations ranging from 0.1 to 20 μM were used to determine the maximum velocity of hydrolysis (V max) for LAP and AP, while for β-GLU slightly lower concentrations (0.1–10 μM) were applied; saturation was generally reached when final concentrations of 10 μM were used. Blanks were prepared using the same water samples, sterilised in autoclave. The increase of fluorescence after 3 hours of incubation at the same temperature measured “*in situ*” (±1 °C) was measured with a Turner TD700 fluorimeter, equipped with specific filters (excitation/emission wavelengths: 380/440 nm for LAP and 365/455 nm for AP and β-GLU). The fluorimeter was calibrated using known concentrations of the standards methylcoumarine (MCA) and methylumbelliferone (MUF), for Leu-MCA and MUF-β-glucoside and MUF-phosphate respectively. The Vmax of the substrate was calculated through the Lineweaver Burke transformation of each substrate concentration plotted *versus* the corresponding velocity of hydrolysis. The Michaelis-Menten constant (K_m_, in μM), as a measure of the affinity or strength of binding between the enzyme and its substrate, was also calculated using the Linewaver-Burk transformation. LAP and β-glu activity rates were further converted into nanograms of C mobilised assuming that 1 nmol of substrate hydrolysed released 72 nanograms of C, while for AP values the conversion took into account that an amount of 31 nanograms of P was released from the hydrolysis of 1 nmol of MUF-phosphate [57].

### 3.4. Other Analysed Parameters

Simultaneous measurements of the following environmental parameters were made: temperature, salinity, dissolved oxygen, nutrients (ammonia, nitrite, nitrate, orthophosphate), autotrophic biomass as Chlorophyll-*a* (Chl-*a*), Temperature, conductivity, dissolved oxygen and fluorescence were measured by a SBE-911 plus conductivity-temperature-depth-Sea Teach fluorometer (CTD-F) profiler, equipped with a Rosette sampler hosting 24 × 10 L Niskin bottles.

Culturable heterotrophic bacterial abundance (CFU/mL) was determined by spreading onto Marine agar plates, incubated at 22 °C for 7 days; total bacterioplankton was counted after DAPI staining and epifluorescence microscope. Chl-*a* content was measured using the acetone extraction method [58]. Particulate Organic Carbon was determined on Whatman GF/F filtering membranes through which appropriate water volumes (generally 500 mL) were concentrated; filters were processed at 980 °C in a Perkin-Elmer CHN-Autoanalyzer 2400, using acetanilide as the standard [59]. Nutrients were determined according to Aminot & Chaussepied method [60], for NH4 concentration, whereas NO2,3 and PO_4_ were measured according to Strickland and Parsons’s method [61].

### 3.5. Statistical Analysis

Statistical differences among the data were assessed by analysis of variance (ANOVA) performed on values logarithmically transformed in order to attain normal distribution; significant relationships between microbial and environmental parameters were calculated by Pearson’s correlation coefficients performed on the dataset.

## 4. Conclusions

The high enzyme activity levels found in this study confirmed the high potential of the microbial community living in coastal ecosystems to play a key role in organic matter decomposition. Enzyme profiles allowed to the microbial community to exploit not only proteins but also organic phosphoesters and cellulose compounds. AP was frequently detected at high activity rates, almost one order of magnitude higher than all the other enzymes; this probably depends on the phytoplankton community, which accounted significantly for the synthesis of this enzyme, as shown by the high specific AP activity rates (*per Chl-a* content) obtained in the studied ecosystems. A great variability characterised the enzyme activity patterns, which were differently expressed in response to the environmental characteristics of the studied environments, *i.e.*, the salinity gradients and trophic inputs, such as in the northern Adriatic basin and in the Gulf of Manfredonia, or the circulation patterns, such as in Straits of Messina. The highest cell-specific LAP and AP activity rates measured both in the Gulf of Milazzo and in the Straits of Messina, in association with low bacterioplankton abundances, suggested that although present in low numbers, bacteria potentially allowed a prompt turnover of the organic matter, faster than that expected.

## Figures and Tables

**Table 1:** Northern Adriatic basin. Mean value ± standard deviation (s.d.), and ranges of variation (minimum–maximum) of enzyme activity rates, Particulate Organic Carbon (POC) and Chlorophyll-*a* (Chl-*a*).

	x ± s.d.	min-max
AP (nM/h)	12.68 ± 18.14	0.35 – 71.32
LAP (nM/h)	5.24 ± 8.17	0.15 – 34.94
β-GLU (nM/h)	2.17 ± 2.86	0.05 – 13.49
POC (μg/L)	8.41 ± 9.93	1.76 – 42.15
Chl-*a* (μg/L)	0.265 ± 0.319	0.05 – 1.78

**Table 2:** Gulf of Manfredonia. Mean value ± standard deviation (s.d.), and ranges of variation (minimum - maximum) of enzyme activity rates and Chlorophyll-*a* (Chl-*a*).

		Transect A	Transect B	Transect C	Transect D
AP	x ± sd	25.79 ± 7.23	28.24 ± 26.11	28.85 ± 21.23	57.93 ± 60.60
nM/h	Min - max	15.55 – 37.75	1.23 – 75.16	0.38 – 93.42	0.41 – 183.81
LAP	x ± sd	26.23 ± 21.08	12.85 ± 9.41	24.33 ± 20.18	8.95 ± 11.38
nM/h	Min - max	3.93 – 61.60	1.63 – 31.11	0.41 – 80.97	0.83 – 38.39
β-GLU	x ± sd	1.082 ± 0.506	0.284 ± 0.222	0.672 ± 0.735	0.489 ± 0.475
nM/h	Min - max	0.561 – 1.703	0.096 – 0.715	0.027 – 2.899	0.003 – 1.428
Chl-*a*	x ± sd	0.263 ± 0.252	0.166 ± 0.111	0.263 ± 0.293	0.278 ± 0.119
μg/L	Min - max	0.041 – 0.600	0.029 – 0.383	0.031 – 1.318	0.189 – 0.507

**Table 3:** Gulf of Milazzo. Mean value ± standard deviation (s.d.), and ranges of variation (minimum–maximum) of enzyme activity rates, culturable heterotrophic bacteria and Chlorophyll-*a* (Chl-*a*) found in December 2002 and February 2003.

December 2002	x ± s.d.	Min–max
LAP (μM/h)	5.36 ± 4.87	0.284–15.36
β-GLU (nM/h)	0.73 ± 0.94	0.054–3.56
AP (nM/h)	244.73 ± 322.73	0.142–867.62
Culturable Heterotrophic bacteria (CFU/mL)	20 ± 19	2–57
Chl-*a* (μg/L)	0.151 ± 0.07	0.015–0.238
**February 2003**	**x ± s.d.**	**min–max**
LAP (nM/h)	5558.1 ± 18724.9	12.31–73089.3
β-GLU (nM/h)	0.006 ± 0.004	0.0011–0.015
AP (nM/h)	62.52 ± 115.60	0.256–463.96
Culturable Heterotrophic bacteria (CFU/mL)	917 ± 678	70 – 1880
Chl-*a* (μg/L)	0.298 ± 0.09	0.065–0.45

**Table 4:** Straits of Messina. Mean value ± standard deviation (s.d.), and ranges of variation (minimum–maximum) of enzyme activity rates, culturable heterotrophic bacteria and Chlorophyll-*a* (Chl-*a*) found at stations Scaletta, Pellaro and Guardia.

	Scaletta	Guardia	Pellaro
LAP (nM/h)			
x ± s.d.	76.52 ± 20.72	1.30 ± 2.12	0.98 ± 1.80
min-max	0.005 – 935.18	0.002 – 9.93	0.0001 – 9.0
β-GLU (nM/h)			
x ± s.d.	12.40 ± 3.19	2.35 ± 7.54	0.97 ± 2.25
min-max	0.005 – 173.29	0.0004 – 38.75	0.0002 – 9.58
AP (nM/h)			
x ± s.d.	121.72 ± 39.1	14.38 ± 37.9	6.38 ± 12.08
min-max	0.005 – 1994.32	0.004 – 143.26	0.0018 – 44.69
Culturable Heterotrophic Bacteria (CFU/mL)			
x ± s.d.	48 ± 10	69 ± 13	50 ± 11
min-max	0 – 340	3 – 260	0 – 472
Chl-*a* (μg/L)			
x ± s.d.	0.109 ± 0.06	0.076 ± 0.04	0.125 ± 0.10
min-max	0.02 – 0.331	0.011 – 0.155	0.037 – 0.492

**Table 5:** Mean amounts of Carbon and Phosphorus potentially released *per hour* and Km values calculated for each enzyme in the examined ecosystems.

	Northern Adriatic	Gulf of Manfredonia	Straits of Messina	Gulf of Milazzo (Dec-2002)	Gulf of Milazzo (Feb-2003)

LAP (ng C/l/h)	0.38	1.40	3.70	0.39	400 × 10^3^
K_m_ LAP (μM)	2.40	0.41	9.51	2.28	268.01
β-GLU (ng C/l/h)	0.16	0.04	0.68	0.05	4 × 10^−4^
K_m_ β-GLU (μM)	0.97	0.29	1.16	0.35	2 × 10^−3^
AP (ng P/l/h)	0.39	1.10	3.35	7.56	1.94
K_m_ AP (μM)	5.38	14.4	3.85	125	33.10

**Table 6:** Reciprocal molar ratios among the different enzyme activities (LAP/β-GLU, LAP/AP) calculated in the examined ecosystems. Cell-specific enzyme activity rates (scaled to total bacterioplankton abundance) are shown; cell-specific AP activity is also scaled to total Chl-*a* content.

	Northern Adriatic	Gulf of Manfredonia	Straits of Messina	Gulf of Milazzo (Dec-2002)	Gulf of Milazzo (Feb-2003)

LAP/β-GLU	2.42	31.58	5.40	7.31	926.3
LAP/AP	0.41	0.55	0.48	0.022	89
Cell-specific LAP (fgC/cell/h)	0.253	10.57	65.55	19	8.72 × 10^3^
Cell-specific β-GLU (fgC/cell/h)	0.105	0.335	12.13	2.60	9.41 × 10^−3^
Cell-specific AP (fgP/cell/h)	0.263	8.27	59.29	374	42.2
Cell-specific AP (μgP/μg Chl-*a*/h)	1.483	4.42	32.64	50.24	6.00
Bacteria (cells/L)	1.49 × 10^9^	1.33 × 10^8^	5.65 × 10^7^	2.03 × 10^7^	4.59 × 10^7^
Chl-*a* (μg/L)	0.265	0.248	0.103	0.151	0.323
